# Supplementary material for: Association of body mass index and waist circumference with long-term mortality risk in 10,370 coronary patients and potential modification by lifestyle and health determinants
Source: PLoS One. 2024 May 31;19(5):e0303329. doi: 10.1371/journal.pone.0303329 (PMC11142547; doi:10.1371/journal.pone.0303329)
Supplement: S1 Table — (DOCX) [file pone.0303329.s001.docx]

**S1 Table. Baseline characteristics of 10,370 patients in the Alpha Omega Cohort & UCC-SMART, stratified for WC^1^.**

|  | **Cohort study** | | | | | |
| --- | --- | --- | --- | --- | --- | --- |
|  | **Alpha Omega Cohort**  **(*n* = 4,837)** | | | **UCC-SMART cohort**  **(*n* = 5,533)** | | |
|  | 1 \| Males: WC < 94; Females: WC < 80  (*n* = 625) | 2 \| Males: WC ≥ 94 - 102; Females: WC ≥ 80 - 88  (*n* = 1,321) | 3 \| Males: WC ≥ 102; Females: WC ≥ 88  (*n* = 2,891) | 1 \| Males: WC < 94; Females: WC < 80  (*n* = 1,618) | 2 \| Males: WC ≥ 94 - 102; Females: WC ≥ 80 - 88  (*n* = 1,684) | 3 \| Males: WC ≥ 102; Females: WC ≥ 88  (*n* = 2,231) |
| **WC, cm** | Males:  89.1 ± 4.4  Females:  75.4 ± 3.4 | Males:  97.9 ± 2.2  Females: 84.3 ± 2.1 | Males:  110.0 ± 7.2  Females: 101.7 ± 9.8 | Males:  87.6 ± 4.8  Females: 80.4 ± 10.1 | Males:  97.5 ± 2.3  Females:  90.7 ± 7.2 | Males:  109.9 ± 7.6  Females:  103.5 ± 10.3 |
| **Age (y)** | 68.8 ± 5.5 | 68.9 ± 5.3 | 69.2 ± 5.7 | 60.3 ± 9.9 | 61.7 ± 9.6 | 62.0 ± 9.2 |
| **Males** | 561 (90) | 1,190 (90) | 2,032 (70) | 1,417 (88) | 1,453 (86) | 1,617 (73) |
| **Smoking** |  |  |  |  |  |  |
| Never | 130 (21) | 214 (16) | 468 (16) | 430 (27) | 403 (24) | 484 (22) |
| Former | 392 (63) | 888 (67) | 1,926 (67) | 804 (50) | 897 (53) | 1,217 (55) |
| Current | 103 (17) | 219 (17) | 497 (17) | 384 (24) | 384 (23) | 530 (24) |
| **Physical activity^2^** |  |  |  |  |  |  |
| Category 1 | 27 (4) | 36 (3) | 214 (7) | 331 (21) | 379 (23) | 675 (31) |
| Category 2 | 219 (35) | 435 (33) | 1,110 (38) | 379 (24) | 422 (26) | 581 (27) |
| Category 3 | 124 (20) | 274 (21) | 607 (21) | 444 (29) | 447 (28) | 492 (23) |
| Category 4 | 255 (41) | 577 (44) | 960 (33) | 397 (24) | 375 (23) | 430 (20) |
| **Alcohol intake^3^** |  |  |  |  |  |  |
| Abstainers | 172 (28) | 314 (24) | 984 (34) | 217 (13) | 241 (14) | 479 (22) |
| Light | 189 (30) | 353 (27) | 694 (24) | 952 (59) | 973 (58) | 1,178 (53) |
| Moderate | 186 (30) | 410 (31) | 759 (26) | 307 (19) | 307 (18) | 362 (16) |
| Heavy | 77 (12) | 241 (18) | 454 (16) | 139 (9) | 158 (9) | 208 (9) |
| **Educational level** |  |  |  |  |  |  |
| Only elementary | 120 (19) | 210 (16) | 673 (24) | 132 (8) | 175 (10) | 347 (16) |
| Low education | 216 (35) | 446 (34) | 1,050 (37) | 237 (15) | 290 (17) | 435 (20) |
| Moderate education | 191 (31) | 453 (35) | 848 (30) | 634 (39) | 744 (44) | 993 (45) |
| High education | 96 (15) | 204 (16) | 297 (10) | 615 (38) | 475 (28) | 456 (20) |
| **Blood pressure, mmHg** |  |  |  |  |  |  |
| Systolic | 139 ± 22 | 142 ± 22 | 142 ± 21 | 134 ± 20 | 137 ± 20 | 139 ± 20 |
| Diastolic | 79 ± 11 | 80 ± 11 | 80 ± 11 | 79 ± 11 | 80 ± 11 | 80 ± 11 |
| **Serum blood lipids, mmol/L^4^** |  |  |  |  |  |  |
| Total cholesterol | 4.6 [4.0, 5.2] | 4.5 [4.0, 5.2] | 4.7 [4.1, 5.3] | 4.3 [3.6, 5.1] | 4.4 [3.8, 5.2] | 4.5 [3.9, 5.3] |
| LDL cholesterol | 2.5 [2.0, 3.0] | 2.5 [2.0, 3.1] | 2.5 [2.0, 3.1] | 2.4 [1.9, 3.1] | 2.5 [2.0, 3.1] | 2.5 [2.0, 3.2] |
| HDL cholesterol | 1.4 [1.1, 1.6] | 1.2 [1.1, 1.5] | 1.2 [1.0, 1.4] | 1.2 [1.0, 1.4] | 1.1 [1.0, 1.3] | 1.1 [0.9, 1.3] |
| Triglycerides | 1.3 [1.0, 1.7] | 1.5 [1.1, 2.1] | 1.8 [1.3, 2.5] | 1.2 [0.9, 1.6] | 1.4 [1.0, 2.0] | 1.6 [1.2, 2.3] |
| **Hs-CRP, mg/L** | 1.2 [0.6, 2.7] | 1.4 [0.7, 3.1] | 2.2 [1.0, 4.3] | 1.3 [0.7, 3.1] | 1.8 [0.9, 3.5] | 2.4 [1.3, 4.6] |
| **Anti-lipid drug use** | 521 (83) | 1,130 (86) | 2,471 (86) | 1,352 (84) | 1,397 (83) | 1,817 (81) |
| **Anti-hypertensive drug use** | 530 (85) | 1,156 (88) | 2,654 (92) | 1,395 (86) | 1,516 (90) | 2,079 (93) |
| **Prevalent diabetes^5^** | 70 (11) | 206 (16) | 738 (26) | 199 (12) | 262 (16) | 578 (26) |
| **DHD-CVD index score^6^** | 89.1 ± 15.6 | 89.1 ± 14.6 | 88.5 ± 14.6 | N/A | N/A | N/A |
| **Self-rated health** |  |  |  |  |  |  |
| Low or moderate | 133 (21) | 243 (17) | 799 (28) | N/A | N/A | N/A |
| Good | 492 (79) | 1,078 (82) | 2,092 (72) | N/A | N/A | N/A |

^1 Values are means ± SD for normally distributed variables, medians [IQRs] for skewed variables or n (%) for categorical or discrete variables; 2 In AOC, defined as 1) no activity, 2) light activity (≤ 3 METs), 3) intermediate activity (moderate or vigorous activity; > 3 METs on >0 to <5 days per week), 4) high activity (moderate or vigorous activity; > 3 METs on >5 days per week), in UCC-SMART defined as quartiles of total physical activity from all activities in MET hours / week; 3 In AOC, defined based on calculated ethanol intake from an FFQ as: abstainers (0 g/d), light (>0-10 g/d in males and > 0-5 g/d in females), moderate (>10-30 g/d in males and >5 – 15 g/d in females) and heavy (>30 g/d in males and > 15 g/d in females, in UCC-SMART defined based on standard drinks (10 g ethanol) per week according to a general questionnaire as: abstainers (0 drinks), light (>0 – 10 drinks), moderate (11-20 drinks) and heavy (>20 drinks). 4 In AOC, measured in a non-fasting state, measured in fasting state in UCC-SMART, measured in a fasting state. 5 In AOC, defined as a self-reported physician diagnosis, use of antidiabetic medication or elevated plasma glucose. In SMART, defined as in AOC, except for self-reported physician diagnosis; 6 DHD-CVD-index score, Dutch Healthy Diet Cardiovascular Disease index.^
