# Supplementary material for: Association of body mass index and waist circumference with long-term mortality risk in 10,370 coronary patients and potential modification by lifestyle and health determinants
Source: PLoS One. 2024 May 31;19(5):e0303329. doi: 10.1371/journal.pone.0303329 (PMC11142547; doi:10.1371/journal.pone.0303329)
Supplement: S2 Table — (DOCX) [file pone.0303329.s002.docx]

**S2 Table. Hazard ratios for BMI in relation to all-cause mortality and CVD mortality in 10,370 CAD patients from AOC and the UCC-SMART.**

|  | Cohort | | | | | |
| --- | --- | --- | --- | --- | --- | --- |
|  | Alpha Omega Cohort | | | UCC-SMART | | |
|  | Categories of BMI | | | Categories of BMI | | |
|  | 1 \| BMI < 25 | 2 \| BMI ≥ 25 - 30 | 3 \| BMI ≥ 30 | 1 \| BMI < 25 | 2 \| BMI ≥ 25 - 30 | 3 \| BMI ≥ 30 |
| **Total population** |  |  |  |  |  |  |
| n | 1,087 | 2,575 | 1,175 | 1,576 | 2,770 | 1,187 |
| Median [IQR] BMI | 23.7 [22.5-24.4] | 27.3 [26.2-28.4] | 32.2 [30.8-34.2] | 23.6 [22.4-24.3] | 27.1 [26.1-28.4] | 32.1 [30.9-34.0] |
| Person-years | 11,574 | 28,770 | 12,855 | 14,884 | 27,059 | 10,949 |
|  |  |  |  |  |  |  |
| **All-cause mortality** |  |  |  |  |  |  |
| Events | 539 | 1,175 | 573 | 380 | 614 | 272 |
| Crude model | 1.15 (1.04, 1.28)^1^ | 1 | 1.09 (0.99, 1.20) | 1.14 (1.00, 1.30) | 1 | 1.12 (0.97, 1.29) |
| Model 1^2^ | 1.13 (1.02, 1.25) | 1 | 1.23 (1.11, 1.36) | 1.06 (0.93, 1.20) | 1 | 1.33 (1.15, 1.54) |
| Model 2^3^ | 1.10 (0.99, 1.22) | 1 | 1.21 (1.09, 1.34) | 1.09 (0.96, 1.25) | 1 | 1.26 (1.09, 1.46) |
| Model 3^4^ | 1.11 (1.01, 1.24) | 1 | 1.15 (1.04, 1.27) | 1.13 (0.99, 1.29) | 1 | 1.21 (1.05, 1.41) |
|  |  |  |  |  |  |  |
| **CVD mortality** |  |  |  |  |  |  |
| Events | 233 | 504 | 273 | 179 | 302 | 129 |
| Crude model | 1.16 (0.99, 1.35) | 1 | 1.20 (1.04, 1.39) | 1.09 (0.91, 1.31) | 1 | 1.07 (0.87, 1.32) |
| Model 1 | 1.12 (0.96, 1.31) | 1 | 1.35 (1.16, 1.57) | 1.01 (0.84, 1.22) | 1 | 1.29 (1.05, 1.59) |
| Model 2 | 1.11 (0.95, 1.29) | 1 | 1.30 (1.12, 1.51) | 1.03 (0.86, 1.25) | 1 | 1.22 (0.99, 1.50) |
| Model 3 | 1.11 (0.95, 1.31) | 1 | 1.26 (1.08, 1.46) | 1.08 (0.99, 1.31) | 1 | 1.17 (0.95, 1.45) |
|  |  |  |  |  |  |  |
| **Males** |  |  |  |  |  |  |
| n | 850 | 2,135 | 798 | 1,232 | 2,350 | 905 |
| Median [IQR] BMI | 23.4 [22.6-24.5] | 27.2 [26.1-28.4] | 32.9 [30.74-33.8] | 23.7 [22.6-24.4] | 27.1 [26.1-28.4] | 31.9 [30.9-33.7] |
| Person-years | 9,033 | 23,874 | 8,852 | 11,838 | 22,937 | 8,219 |
|  |  |  |  |  |  |  |
| **All-cause mortality** |  |  |  |  |  |  |
| Events | 422 | 957 | 376 | 294 | 531 | 215 |
| Crude model | 1.18 (1.05, 1.32) | 1 | 1.06 (0.94, 1.20) | 1.08 (0.94, 1.24) | 1 | 1.15 (0.99, 1.35) |
| Model 1 | 1.12 (1.01, 1.27) | 1 | 1.29 (1.15, 1.46) | 0.96 (0.83, 1.11) | 1 | 1.41 (1.20, 1.65) |
| Model 2 | 1.13 (1.00, 1.26) | 1 | 1.25 (1.11, 1.41) | 1.01 (0.87, 1.16) | 1 | 1.31 (1.11, 1.54) |
| Model 3 | 1.13 (1.01, 1.27) | 1 | 1.19 (1.05, 1.34) | 1.03 (0.89, 1.20) | 1 | 1.29 (1.09, 1.52) |
|  |  |  |  |  |  |  |
| **CVD mortality** |  |  |  |  |  |  |
| Events | 194 | 392 | 177 | 138 | 261 | 107 |
| Crude model | 1.32 (1.11, 1.56) | 1 | 1.22 (1.02, 1.46) | 1.03 (0.84, 1.27) | 1 | 1.17 (0.93, 1.46) |
| Model 1 | 1.26 (1.06, 1.49) | 1 | 1.49 (1.25, 1.79) | 0.91 (0.74, 1.12) | 1 | 1.43 (1.14, 1.79) |
| Model 2 | 1.26 (1.07, 1.51) | 1 | 1.42 (1.19, 1.70) | 0.94 (0.76, 1.16) | 1 | 1.34 (1.06, 1.68) |
| Model 3 | 1.27 (1.06, 1.51) | 1 | 1.36 (1.14, 1.63) | 0.98 (0.79, 1.21) | 1 | 1.33 (1.05, 1.67) |
|  |  |  |  |  |  |  |
| **Females** |  |  |  |  |  |  |
| n | 237 | 440 | 377 | 344 | 420 | 282 |
| Median [IQR] BMI | 23.2 [21.8-24.3] | 27.5 [26.3-28.7] | 32.6 [31.1-35.1] | 23.0 [21.5-24.0] | 27.0 [26.0-28.3] | 32.5 [31.0-35.1] |
| Person-years | 2,542 | 4,895 | 4,004 | 3,046 | 4,122 | 2,730 |
|  |  |  |  |  |  |  |
| **All-cause mortality** |  |  |  |  |  |  |
| Events | 117 | 218 | 197 | 86 | 83 | 57 |
| Crude model | 1.03 (0.83, 1.29) | 1 | 1.09 (0.90, 1.33) | 1.50 (1.11, 2.03) | 1 | 1.05 (0.75, 1.47) |
| Model 1 | 1.08 (0.86, 1.35) | 1 | 1.10 (0.91, 1.33) | 1.57 (1.16, 2.13) | 1 | 1.15 (0.82, 1.62) |
| Model 2 | 1.00 (0.80, 1.26) | 1 | 1.10 (0.90, 1.33) | 1.51 (1.10, 2.08) | 1 | 1.08 (0.77, 1.53) |
| Model 3 | 1.03 (0.82, 1.30) | 1 | 1.05 (0.86, 1.28) | 1.60 (1.17, 2.20) | 1 | 0.99 (0.70, 1.40) |
|  |  |  |  |  |  |  |
| **CVD mortality** |  |  |  |  |  |  |
| Events | 39 | 112 | 96 | 41 | 41 | 22 |
| Crude model | 0.67 (0.46, 0.96) | 1 | 1.01 (0.77, 1.33) | 1.42 (0.92, 2.20) | 1 | 0.81 (0.48, 1.37) |
| Model 1 | 0.69 (0.48, 1.00) | 1 | 1.03 (0.78, 1.35) | 1.49 (0.97, 2.30) | 1 | 0.89 (0.53, 1.50) |
| Model 2 | 0.66 (0.46, 0.96) | 1 | 1.00 (0.76, 1.32) | 1.49 (0.95, 2.33) | 1 | 0.84 (0.49, 1.43) |
| Model 3 | 0.70 (0.48, 1.01) | 1 | 0.96 (0.73, 1.28) | 1.63 (1.04, 2.56) | 1 | 0.72 (0.42, 1.23) |

^1^ Hazard ratio (95% confidence interval) obtained from Cox proportional hazards models (all such values), using the middle category as the reference; ^2^Adjusted for age and sex, not adjusted for sex in sex-stratified results; ^3^Adjusted as model 1, plus for smoking status, physical activity, educational level and alcohol intake, this model was used as the main model; ^4^Adjusted as model 2, plus for diabetes, systolic blood pressure, LDL-cholesterol and hs-CRP.
