# Supplementary material for: Association of body mass index and waist circumference with long-term mortality risk in 10,370 coronary patients and potential modification by lifestyle and health determinants
Source: PLoS One. 2024 May 31;19(5):e0303329. doi: 10.1371/journal.pone.0303329 (PMC11142547; doi:10.1371/journal.pone.0303329)
Supplement: S3 Table — (DOCX) [file pone.0303329.s003.docx]

**S3 Table. Hazard ratios for WC in relation to all-cause mortality and CVD mortality in 10,370 CAD patients from AOC and the UCC-SMART.**

|  | Cohort | | | | | |
| --- | --- | --- | --- | --- | --- | --- |
|  | Alpha Omega Cohort | | | UCC-SMART | | |
|  | Categories of WC | | | Categories of WC | | |
|  | 1 \| Males: WC < 94; Females: WC < 80 | 2 \| Males: WC ≥ 94 - 102; Females: WC ≥ 80 - 88 | 3 \| Males: WC ≥ 102; Females: WC ≥ 88 | 1 \| Males: WC < 94; Females: WC < 80 | 2 \| Males: WC ≥ 94 - 102; Females: WC ≥ 80 - 88 | 3 \| Males: WC ≥ 102; Females: WC ≥ 88 |
| **Total population** |  |  |  |  |  |  |
| n | 625 | 1321 | 2891 | 1,618 | 1,684 | 2,231 |
| Median [IQR] WC | Males:  90 [88 - 92]  Females:  76 [73 - 78] | Males:  98 [96 - 100]  Females:  85 [83 - 86] | Males:  108 [104 - 113]  Females:  100 [94 - 107] | Males:  89 [85 - 91]  Females:  74 [71 - 77] | Males:  98 [96 - 100]  Females:  84 [82 - 86] | Males:  108 [104 - 113]  Females:  97 [92 - 104] |
| Person-years | 6,955 | 15,014 | 31,230 | 15,673 | 16,610 | 20,609 |
|  |  |  |  |  |  |  |
| **All-cause mortality** |  |  |  |  |  |  |
| Events | 275 | 574 | 1,438 | 321 | 357 | 588 |
| Crude model | 1.04 (0.90, 1.20)^1^ | 1 | 1.22 (1.11, 1.35) | 0.96 (0.83, 1.12) | 1 | 1.35 (1.19, 1.54) |
| Model 1^2^ | 1.05 (0.91, 1.21) | 1 | 1.22 (1.11, 1.35) | 1.03 (0.88, 1.19) | 1 | 1.38 (1.20, 1.57) |
| Model 2^3^ | 1.01 (0.88, 1.17) | 1 | 1.16 (1.05, 1.28) | 1.08 (0.93, 1.25) | 1 | 1.26 (1.09, 1.44) |
| Model 3^4^ | 1.02 (0.88, 1.18) | 1 | 1.13 (1.02, 1.24) | 1.11 (0.96, 1.30) | 1 | 1.23 (1.07, 1.41) |
|  |  |  |  |  |  |  |
| **CVD mortality** |  |  |  |  |  |  |
| Events | 131 | 234 | 645 | 150 | 171 | 289 |
| Crude model | 1.22 (0.98, 1.50) | 1 | 1.35 (1.16, 1.56) | 0.94 (0.75, 1.16) | 1 | 1.38 (1.14, 1.67) |
| Model 1 | 1.22 (0.99, 1.51) | 1 | 1.33 (1.14, 1.55) | 1.00 (0.80, 1.24) | 1 | 1.42 (1.17, 1.72) |
| Model 2 | 1.18 (0.95, 1.46) | 1 | 1.24 (1.07, 1.45) | 1.05 (0.84, 1.31) | 1 | 1.30 (1.07, 1.58) |
| Model 3 | 1.21 (0.98, 1.50) | 1 | 1.21 (1.04, 1.41) | 1.09 (0.88, 1.37) | 1 | 1.26 (1.04, 1.53) |
|  |  |  |  |  |  |  |
| **Males** |  |  |  |  |  |  |
| n | 561 | 1,190 | 2,032 | 1,417 | 1,453 | 1,617 |
| Median [IQR] WC | 90 [88 - 92] | 98 [96 - 98] | 108 [104 - 114] | 89 [85 - 91] | 98 [96 - 100] | 108 [104 - 113] |
| Person-years | 6,267 | 13,478 | 22,014 | 13,791 | 14,385 | 14,819 |
|  |  |  |  |  |  |  |
| **All-cause mortality** |  |  |  |  |  |  |
| Crude model | 247 | 521 | 987 | 291 | 317 | 432 |
| Model 1 | 1.03 (0.88, 1.19) | 1 | 1.06 (0.94, 1.20) | 0.96 (0.82, 1.13) | 1 | 1.36 (1.18, 1.57) |
| Model 2 | 1.02 (0.88, 1.18) | 1 | 1.19 (1.07, 1.34) | 1.00 (0.86, 1.18) | 1 | 1.36 (1.18, 1.58) |
| Model 3 | 1.00 (0.87, 1.17) | 1 | 1.13 (1.02, 1.26) | 1.07 (0.91, 1.25) | 1 | 1.26 (1.08, 1.46) |
| Crude model | 1.02 (0.87, 1.18) | 1 | 1.10 (0.99, 1.23) | 1.10 (0.93, 1.29) | 1 | 1.25 (1.08, 1.45) |
|  |  |  |  |  |  |  |
| **CVD mortality** |  |  |  |  |  |  |
| Events | 119 | 214 | 430 | 138 | 155 | 213 |
| Crude model | 1.20 (0.96, 1.50) | 1 | 1.24 (1.05, 1.46) | 0.93 (0.74, 1.18) | 1 | 1.37 (1.11, 1.68) |
| Model 1 | 1.18 (0.95, 1.48) | 1 | 1.25 (1.07, 1.48) | 0.97 (0.77, 1.22) | 1 | 1.37 (1.11, 1.68) |
| Model 2 | 1.17 (0.93, 1.46) | 1 | 1.18 (1.00, 1.39) | 1.04 (0.82, 1.31) | 1 | 1.28 (1.04, 1.59) |
| Model 3 | 1.20 (0.96, 1.51) | 1 | 1.16 (0.98, 1.37) | 1.07 (0.85, 1.36) | 1 | 1.27 (1.03, 1.57) |
|  |  |  |  |  |  |  |
| **Females** |  |  |  |  |  |  |
| n | 64 | 131 | 859 | 201 | 231 | 614 |
| Median [IQR] WC | 76 [73 - 78] | 85 [83 - 86] | 100 [94.0 - 107] | 74 [71 -77] | 84 [82 - 86] | 97 [92 - 104] |
| Person-years | 687 | 1,537 | 9,216 | 1,883 | 2,225 | 5,789 |
|  |  |  |  |  |  |  |
| **All-cause mortality** |  |  |  |  |  |  |
| Events | 28 | 53 | 451 | 30 | 40 | 156 |
| Crude model | 1.21 (0.76, 1.92) | 1 | 1.48 (1.11, 1.97) | 0.90 (0.56, 1.44) | 1 | 1.44 (1.01, 2.03) |
| Model 1 | 1.32 (0.84, 2.09) | 1 | 1.47 (1.10, 1.96) | 1.17 (0.73, 1.89) | 1 | 1.43 (1.01, 2.04) |
| Model 2 | 1.09 (0.69, 1.75) | 1 | 1.37 (1.03, 1.83) | 1.14 (0.70, 1.86) | 1 | 1.23 (0.86, 1.76) |
| Model 3 | 1.16 (0.73, 1.87) | 1 | 1.35 (1.01, 1.81) | 1.22 (0.75, 2.00) | 1 | 1.12 (0.78, 1.62) |
|  |  |  |  |  |  |  |
| **CVD mortality** |  |  |  |  |  |  |
| Crude model | 12 | 20 | 215 | 12 | 16 | 76 |
| Model 1 | 1.39 (0.68, 2.85) | 1 | 1.89 (0.19, 2.98) | 0.90 (0.43, 1.91) | 1 | 1.78 (1.03, 3.05) |
| Model 2 | 1.55 (0.76, 3.17) | 1 | 1.88 (1.19, 2.97) | 1.16 (0.54, 2.45) | 1 | 1.77 (1.03, 3.05) |
| Model 3 | 1.46 (0.70, 3.03) | 1 | 1.78 (1.12, 2.82) | 1.14 (0.53, 2.42) | 1 | 1.43 (0.83, 2.49) |
| Crude model | 1.53 (0.73, 3.21) | 1 | 1.69 (1.06, 2.70) | 1.30 (0.61, 2.81) | 1 | 1.24 (0.71, 2.18) |

^1^ Hazard ratio (95% confidence interval) obtained from Cox proportional hazards models (all such values), using the middle category as the reference; ^2^Adjusted for age and sex, not adjusted for sex in sex-stratified results; ^3^Adjusted as model 1, plus for smoking status, physical activity, educational level and alcohol intake, this model was used as the main model; ^4^Adjusted as model 2, plus for diabetes, systolic blood pressure, LDL-cholesterol and hs-CRP.
