# Supplementary material for: Association of body mass index and waist circumference with long-term mortality risk in 10,370 coronary patients and potential modification by lifestyle and health determinants
Source: PLoS One. 2024 May 31;19(5):e0303329. doi: 10.1371/journal.pone.0303329 (PMC11142547; doi:10.1371/journal.pone.0303329)
Supplement: S4 Table — (DOCX) [file pone.0303329.s004.docx]

**S4 Table. Hazard ratios for BMI in relation to all-cause mortality and CVD mortality in 9,620 CAD patients from AOC and the UCC-SMART excluding the first two years of follow-up.**

|  | Pooled analysis | | |
| --- | --- | --- | --- |
|  | Categories of BMI | | |
|  | 1 \| BMI < 25 | 2 \| BMI ≥ 25 - 30 | 3 \| BMI ≥ 30 |
| **Total population** |  |  |  |
| n | 2,457 | 4,984 | 2,179 |
| Person-years | 24,174 | 50,452 | 21,370 |
|  |  |  |  |
| **All-cause mortality** |  |  |  |
| Events | 829 | 1,645 | 1,359 |
| Crude model | 1.14 (1.05, 1.23)^1^ | 1 | 1.09 (1.00, 1.18) |
| Model 1^2^ | 1.09 (1.00, 1.18) | 1 | 1.27 (1.12, 1.44) |
| Model 2^3^ | 1.08 (1.00, 1.18) | 1 | 1.22 (1.12, 1.32) |
|  |  |  |  |
| **CVD mortality** |  |  |  |
| Events | 361 | 722 | 357 |
| Crude model | 1.12 (0.98, 1.27) | 1 | 1.16 (1.02, 1.33) |
| Model 1 | 1.06 (0.94, 1.20) | 1 | 1.34 (1.18, 1.53) |
| Model 2 | 1.06 (0.93, 1.20) | 1 | 1.29 (1.14, 1.47) |

^1^ Pooled hazard ratio (95% confidence interval) obtained from Cox proportional hazards models (all such values), using the middle category as the reference, and random effects meta-analysis; ^2^Adjusted for age and sex; ^3^Adjusted as model 1, plus for smoking status, physical activity, educational level and alcohol intake.
