# Supplementary material for: Association of body mass index and waist circumference with long-term mortality risk in 10,370 coronary patients and potential modification by lifestyle and health determinants
Source: PLoS One. 2024 May 31;19(5):e0303329. doi: 10.1371/journal.pone.0303329 (PMC11142547; doi:10.1371/journal.pone.0303329)
Supplement: S5 Table — (DOCX) [file pone.0303329.s005.docx]

**S5 Table. Hazard ratios for BMI in relation to CVD and all-cause mortality in 8,337 CAD patients from AOC and the UCC-SMART excluding the first five years of follow-up.**

|  | Pooled analysis | | |
| --- | --- | --- | --- |
|  | Categories of BMI | | |
|  | 1 \| BMI < 25 | 2 \| BMI ≥ 25 - 30 | 3 \| BMI ≥ 30 |
| **Total population** |  |  |  |
| n | 2,094 | 4,368 | 1,875 |
| Person-years | 20,353 | 41,865 | 17,434 |
|  |  |  |  |
| **All-cause mortality** |  |  |  |
| Events | 643 | 1,316 | 606 |
| Crude model | 1.11 (1.01, 1.23)^1^ | 1 | 1.08 (0.98, 1.19) |
| Model 1^2^ | 1.07 (0.97, 1.17) | 1 | 1.26 (1.14, 1.39) |
| Model 2^3^ | 1.07 (0.97, 1.17) | 1 | 1.23 (1.11, 1.36) |
|  |  |  |  |
| **CVD mortality** |  |  |  |
| Events | 380 | 375 | 199 |
| Crude model | 1.09 (0.94, 1.26) | 1 | 1.09 (0.86, 1.37) |
| Model 1 | 1.04 (0.90, 1.20) | 1 | 1.28 (1.11, 1.48) |
| Model 2 | 1.03 (0.90, 1.20) | 1 | 1.23 (1.02, 1.47) |

^1^ Pooled hazard ratio (95% confidence interval) obtained from Cox proportional hazards models (all such values), using the middle category as the reference, and random effects meta-analysis; ^2^Adjusted for age and sex; ^3^Adjusted as model 1, plus for smoking status, physical activity, educational level and alcohol intake.
