# Supplementary material for: Association of body mass index and waist circumference with long-term mortality risk in 10,370 coronary patients and potential modification by lifestyle and health determinants
Source: PLoS One. 2024 May 31;19(5):e0303329. doi: 10.1371/journal.pone.0303329 (PMC11142547; doi:10.1371/journal.pone.0303329)
Supplement: S6 Table — (DOCX) [file pone.0303329.s006.docx]

**S6 Table.** **Hazard ratios for WC in relation to all-cause mortality and CVD mortality in 9,620 CAD patients from AOC and the UCC-SMART excluding the first two years of follow-up.**

|  | Pooled analysis | | |
| --- | --- | --- | --- |
|  | Categories of WC | | |
|  | 1 \| Males: WC < 94; Females: WC < 80 | 2 \| Males: WC ≥ 94 - 102; Females: WC ≥ 80 - 88 | 3 \| Males: WC ≥ 102; Females: WC ≥ 88 |
| **Total population** |  |  |  |
| n | 2,046 | 2,818 | 4,756 |
| Person-years | 21,239 | 28,862 | 45,895 |
|  |  |  |  |
| **All-cause mortality** |  |  |  |
| Events | 540 | 861 | 1,837 |
| Crude model | 0.99 (0.88, 1.10)^1^ | 1 | 1.27 (1.12, 1.43) |
| Model 1^2^ | 1.02 (0.91, 1.14) | 1 | 1.28 (1.11, 1.46) |
| Model 2^3^ | 1.02 (0.90, 1.15) | 1 | 1.19 (1.06, 1.33) |
|  |  |  |  |
| **CVD mortality** |  |  |  |
| Events | 249 | 367 | 824 |
| Crude model | 1.05 (0.89, 1.24) | 1 | 1.33 (1.18, 1.51) |
| Model 1 | 1.09 (0.93, 1.28) | 1 | 1.33 (1.17, 1.51) |
| Model 2 | 1.09 (0.93, 1.28) | 1 | 1.24 (1.09, 1.41) |

^1^ Pooled hazard ratio (95% confidence interval) obtained from Cox proportional hazards models (all such values), using the middle category as the reference, and random effects meta-analysis; ^2^Adjusted for age and sex; ^3^Adjusted as model 1, plus for smoking status, physical activity, educational level and alcohol intake.
