# Supplementary material for: Association of body mass index and waist circumference with long-term mortality risk in 10,370 coronary patients and potential modification by lifestyle and health determinants
Source: PLoS One. 2024 May 31;19(5):e0303329. doi: 10.1371/journal.pone.0303329 (PMC11142547; doi:10.1371/journal.pone.0303329)
Supplement: S8 Table — (DOCX) [file pone.0303329.s008.docx]

**S8 Table.** **Hazard ratios for BMI in relation to all-cause mortality and CVD mortality in 4,837 CAD patients from AOC excluding patients with cancer.**

|  | Categories of BMI | | |
| --- | --- | --- | --- |
|  | 1 \| BMI < 25 | 2 \| BMI ≥ 25 - 30 | 3 \| BMI ≥ 30 |
| **Total population** |  |  |  |
| n | 958 | 2,274 | 1,025 |
| Person-years | 10,364 | 25,763 | 11,408 |
|  |  |  |  |
| **All-cause mortality** |  |  |  |
| Events | 458 | 994 | 483 |
| Crude model | 1.16 (1.04, 1.30)^1^ | 1 | 1.10 (0.99, 1.23) |
| Model 1^2^ | 1.14 (1.02, 1.27) | 1 | 1.26 (1.13, 1.41) |
| Model 2^3^ | 1.12 (1.00, 1.25) | 1 | 1.25 (1.11, 1.39) |
|  |  |  |  |
| **CVD mortality** |  |  |  |
| Events | 200 | 435 | 236 |
| Crude model | 1.15 (0.98, 1.37) | 1 | 1.23 (1.05, 1.44) |
| Model 1 | 1.13 (0.95, 1.33) | 1 | 1.40 (1.19, 1.65) |
| Model 2 | 1.11 (0.94, 1.32) | 1 | 1.36 (1.15, 1.59) |

^1^ Pooled hazard ratio (95% confidence interval) obtained from Cox proportional hazards models (all such values), using the middle category as the reference, and random effects meta-analysis; ^2^Adjusted for age and sex; ^3^Adjusted as model 1, plus for smoking status, physical activity, educational level and alcohol intake.
