# Supplementary material for: Association of body mass index and waist circumference with long-term mortality risk in 10,370 coronary patients and potential modification by lifestyle and health determinants
Source: PLoS One. 2024 May 31;19(5):e0303329. doi: 10.1371/journal.pone.0303329 (PMC11142547; doi:10.1371/journal.pone.0303329)
Supplement: S9 Table — (DOCX) [file pone.0303329.s009.docx]

**S9 Table.** **Hazard ratios for WC in relation to all-cause mortality and CVD mortality in 4,837 CAD patients from AOC excluding patients with cancer.**

|  | Categories of BMI | | |
| --- | --- | --- | --- |
|  | 1 \| Males: WC < 94; Females: WC < 80 | 2 \| Males: WC ≥ 94 - 102; Females: WC ≥ 80 - 88 | 3 \| Males: WC ≥ 102; Females: WC ≥ 88 |
| **Total population** |  |  |  |
| n | 558 | 1,163 | 2,536 |
| Person-years | 6,311 | 13,385 | 27,840 |
|  |  |  |  |
| **All-cause mortality** |  |  |  |
| Events | 237 | 482 | 1,216 |
| Crude model | 1.05 (0.90, 1.23)^1^ | 1 | 1.24 (1.11, 1.38) |
| Model 1^2^ | 1.08 (0.92, 1.26) | 1 | 1.24 (1.12, 1.39) |
| Model 2^3^ | 1.02 (0.87, 1.19) | 1 | 1.16 (1.04, 1.30) |
|  |  |  |  |
| **CVD mortality** |  |  |  |
| Events | 113 | 197 | 561 |
| Crude model | 1.22 (0.97, 1.54) | 1 | 1.39 (1.18, 1.64) |
| Model 1 | 1.25 (1.00, 1.58) | 1 | 1.38 (1.17, 1.63) |
| Model 2 | 1.21 (0.96, 1.52) | 1 | 1.29 (1.09, 1.52) |

^1^ Pooled hazard ratio (95% confidence interval) obtained from Cox proportional hazards models (all such values), using the middle category as the reference, and random effects meta-analysis; ^2^Adjusted for age and sex; ^3^Adjusted as model 1, plus for smoking status, physical activity, educational level and alcohol intake.
