# Supplementary figures and images for: Association of body mass index and waist circumference with long-term mortality risk in 10,370 coronary patients and potential modification by lifestyle and health determinants
Source: PLoS One. 2024 May 31;19(5):e0303329. doi: 10.1371/journal.pone.0303329 (PMC11142547; doi:10.1371/journal.pone.0303329)

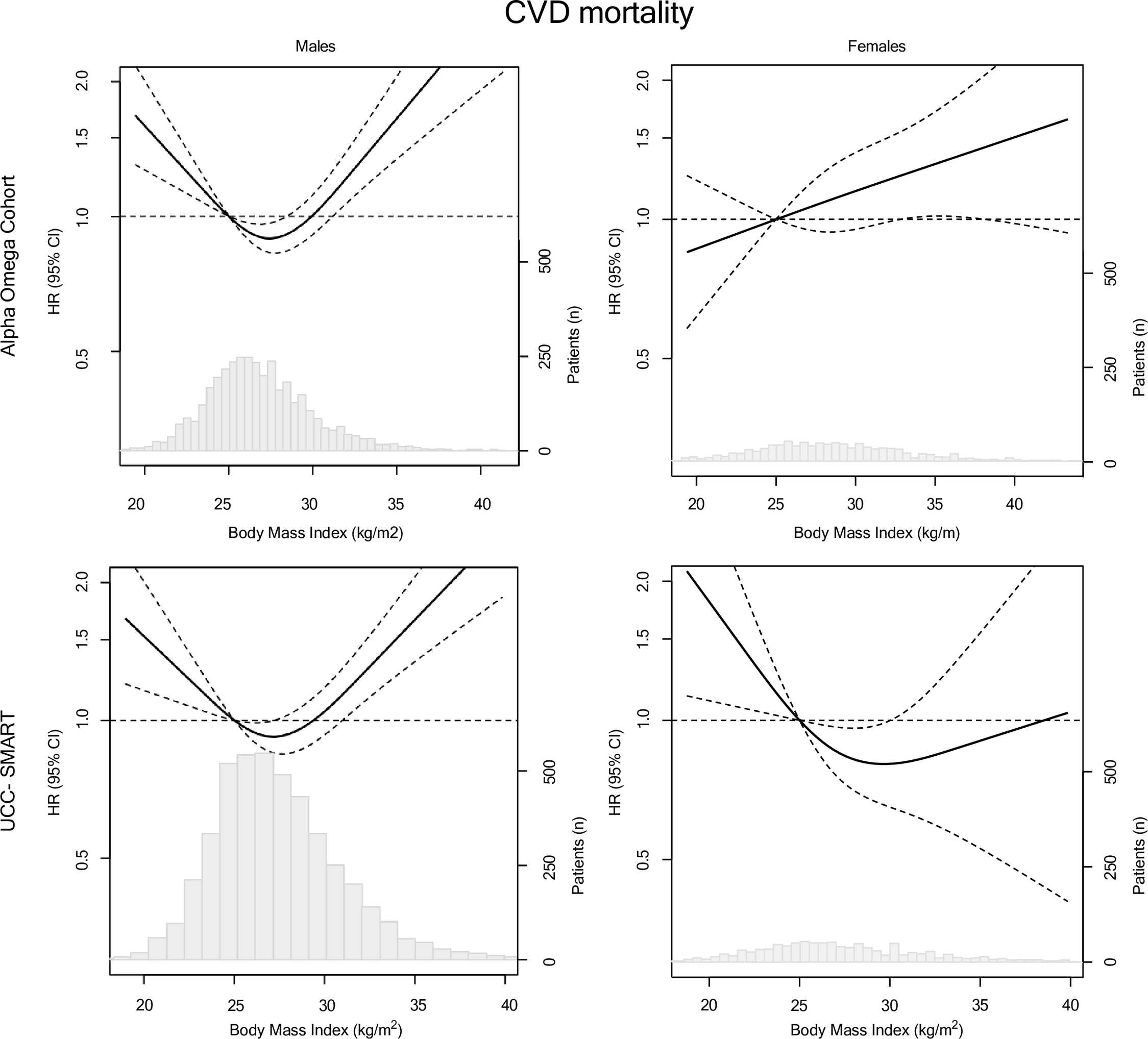

Supplement: S1 Fig — Lines are restricted cubic splines, showing 3 knots at the 10th, 50th and 90th percentiles. The y-axis shows the predicted HRs for mortality for any value of BMI, compared to the reference, set at 25 kg/m2. Results are presented for model 2. (TIF) [file pone.0303329.s010.tif]

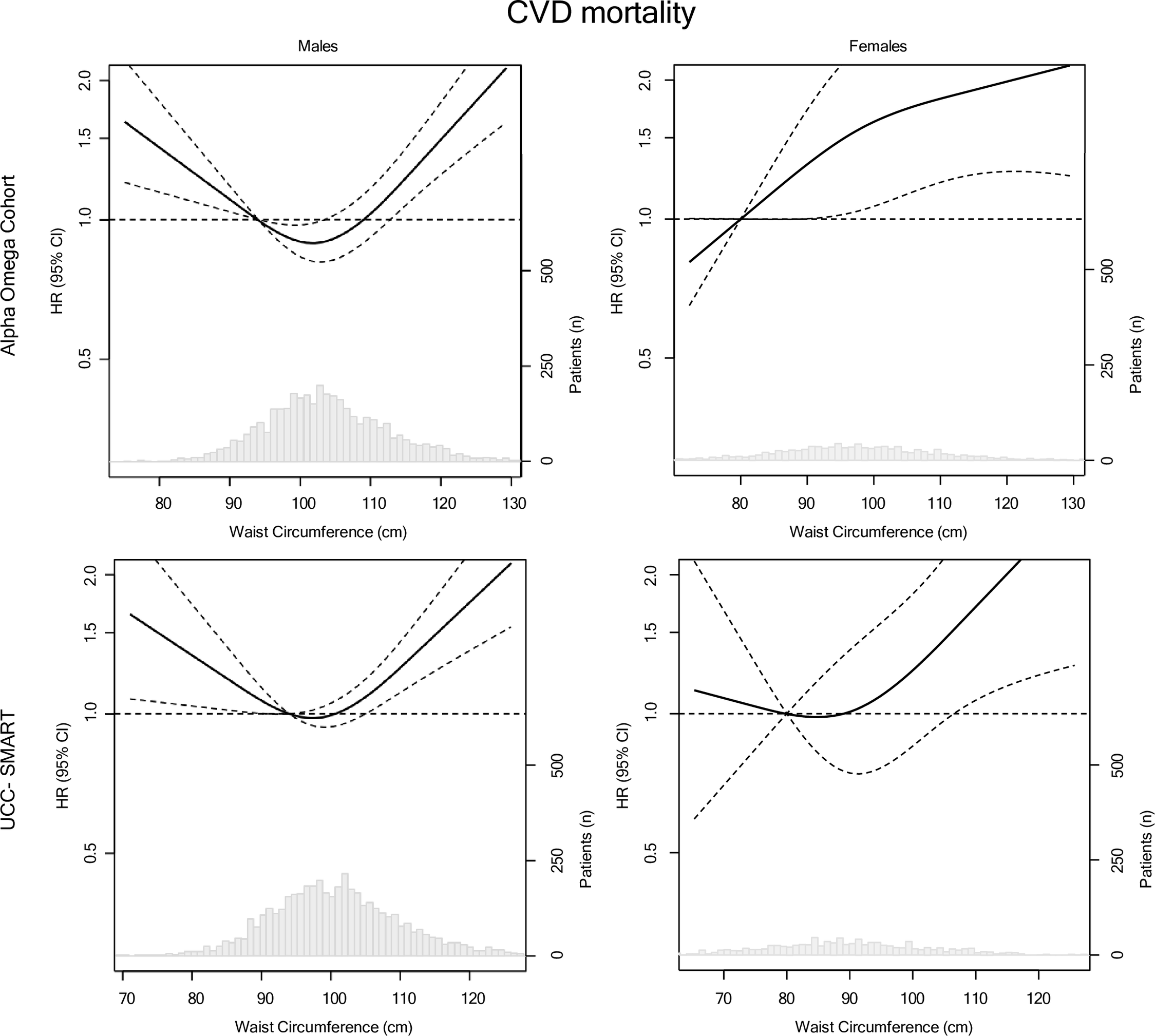

Supplement: S2 Fig — Lines are restricted cubic splines, showing 3 knots at the 10th, 50th and 90th percentiles. The y-axis shows the predicted HRs for mortality for any value of WC, compared to the reference, set at 94 cm for males and 80 cm for females. Results are presented for model 2. (TIF) [file pone.0303329.s011.tif]

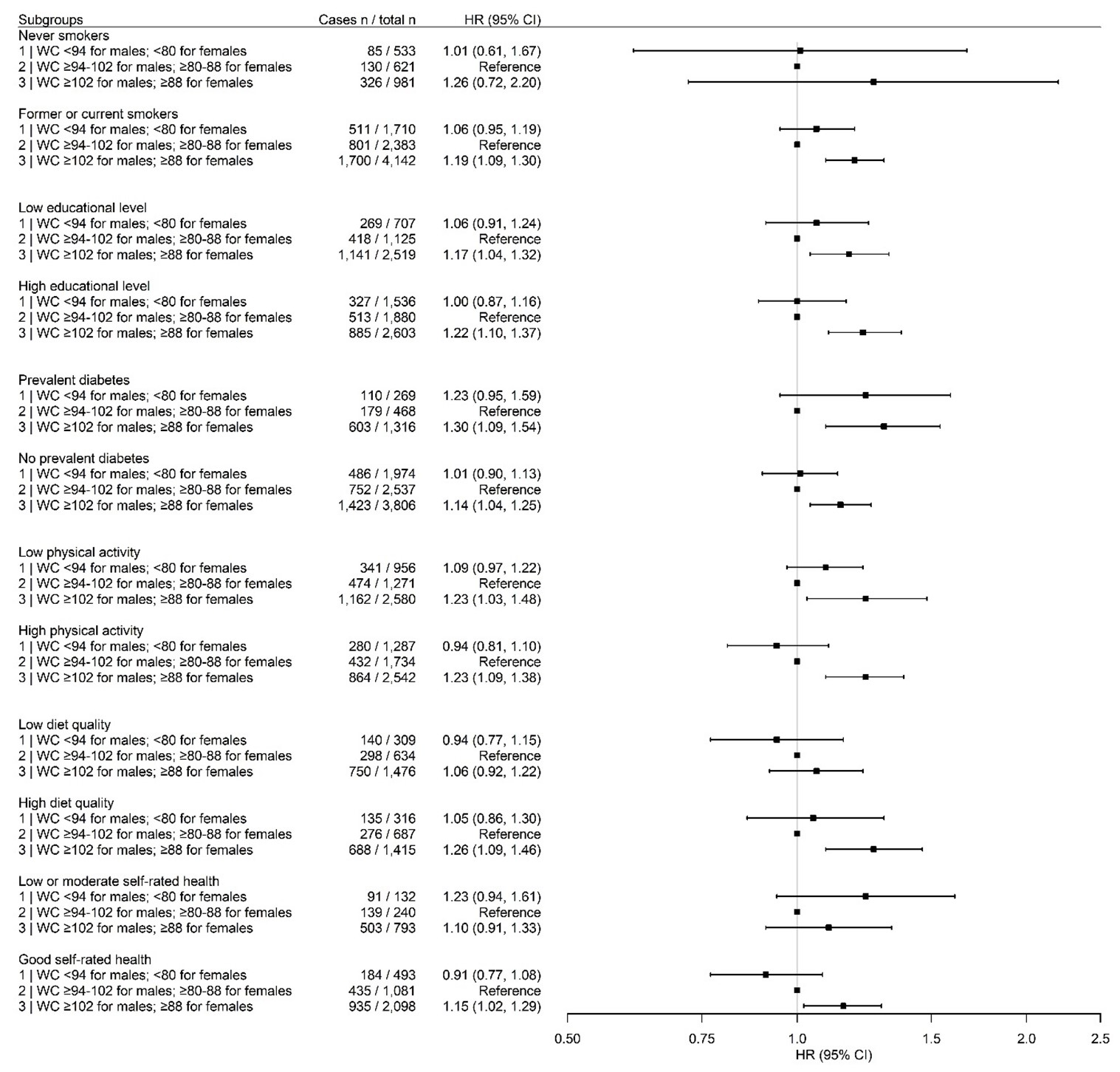

Supplement: S3 Fig — Associations were adjusted according to variables in model 2 unless for variable stratified for. Results for diet quality and self-rated health only available from AOC. (TIF) [file pone.0303329.s012.tif]

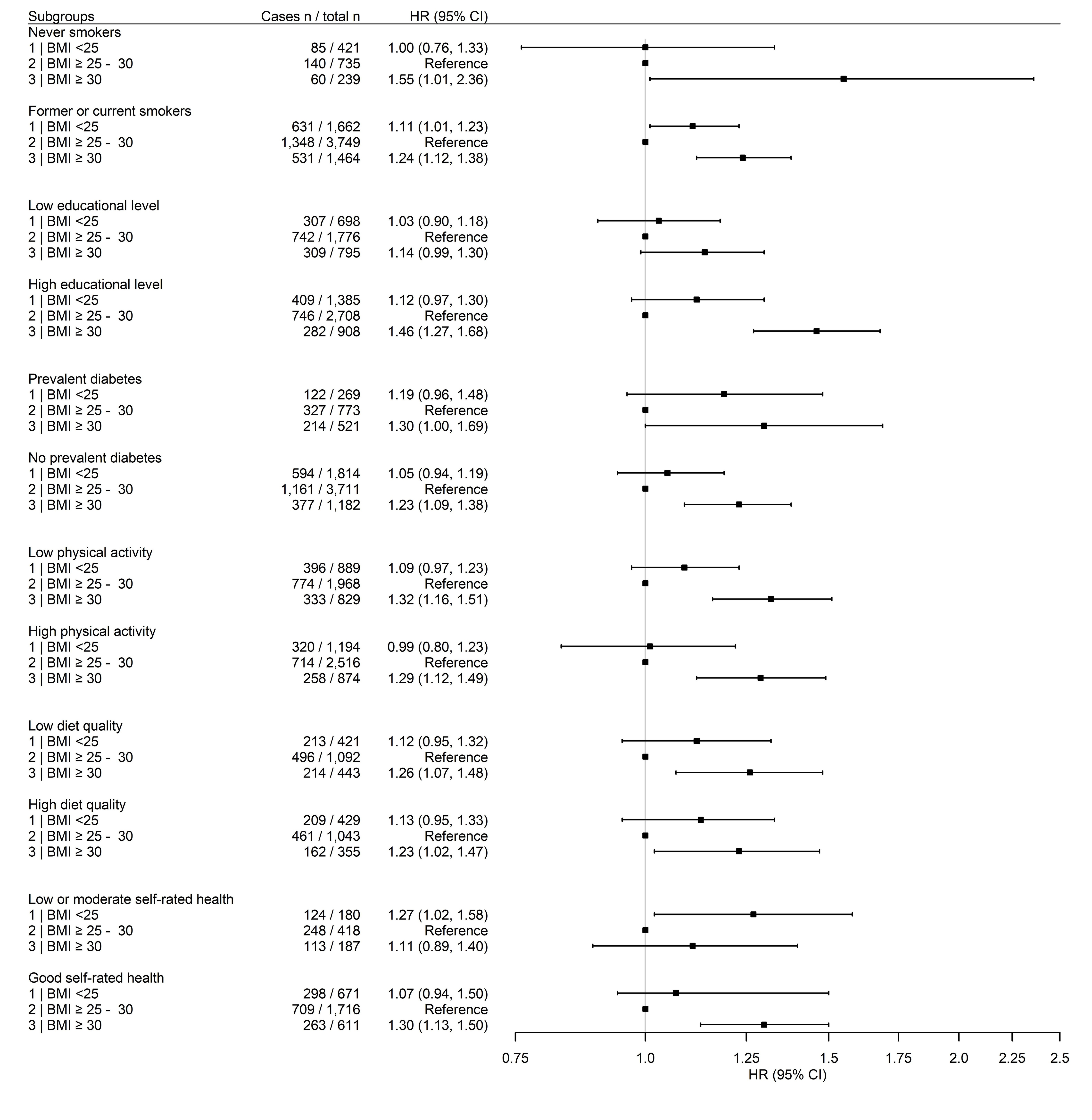

Supplement: S4 Fig — Associations were adjusted according to variables in model 2 unless for variable stratified for. Results for diet quality and self-rated health only available from AOC. (TIF) [file pone.0303329.s013.tif]

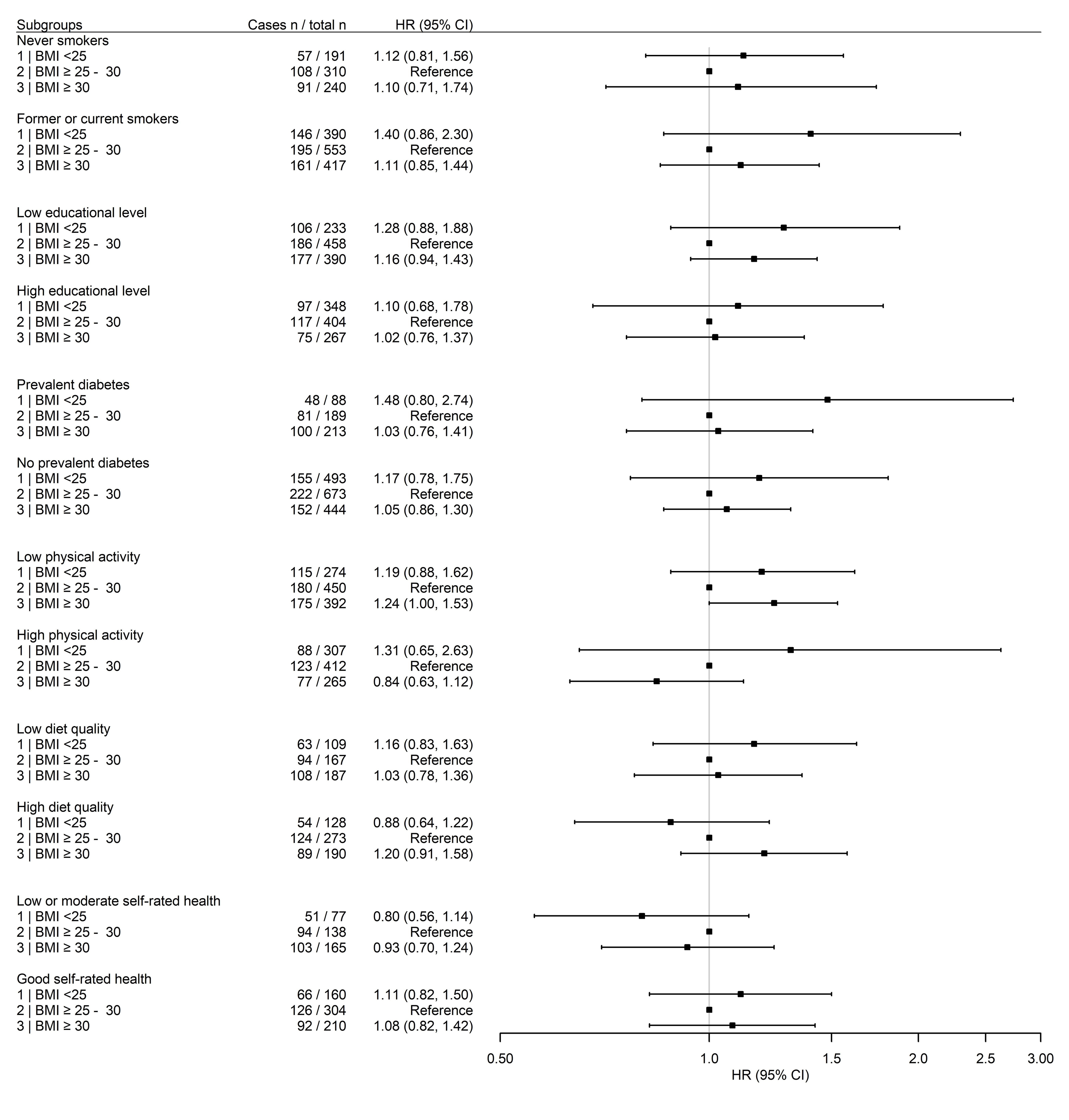

Supplement: S5 Fig — Associations were adjusted according to variables in model 2 unless for variable stratified for. Results for diet quality and self-rated health only available from AOC. (TIF) [file pone.0303329.s014.tif]

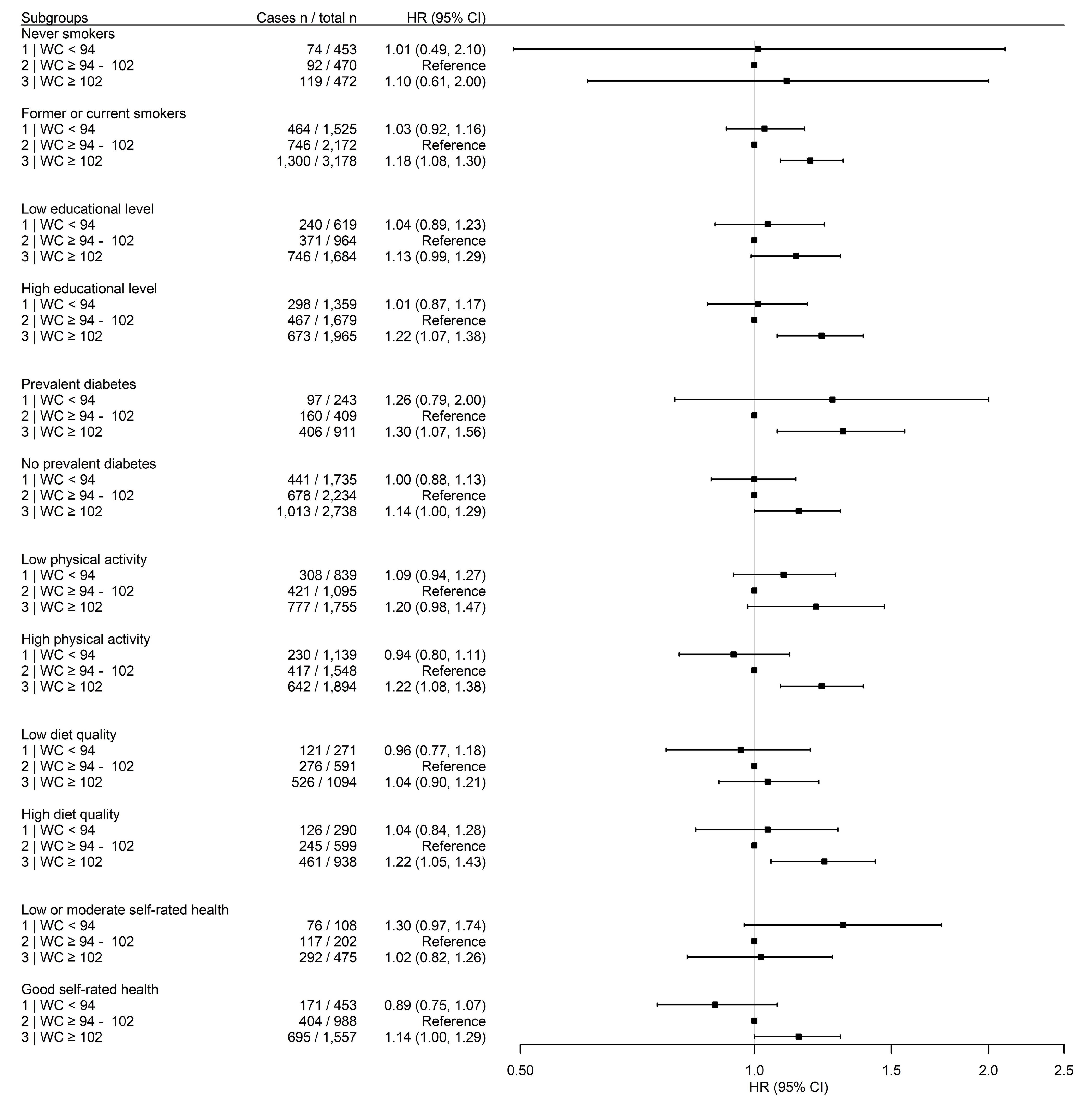

Supplement: S6 Fig — Associations were adjusted according to variables in model 2 unless for variable stratified for. Results for diet quality and self-rated health only available from AOC. (TIF) [file pone.0303329.s015.tif]

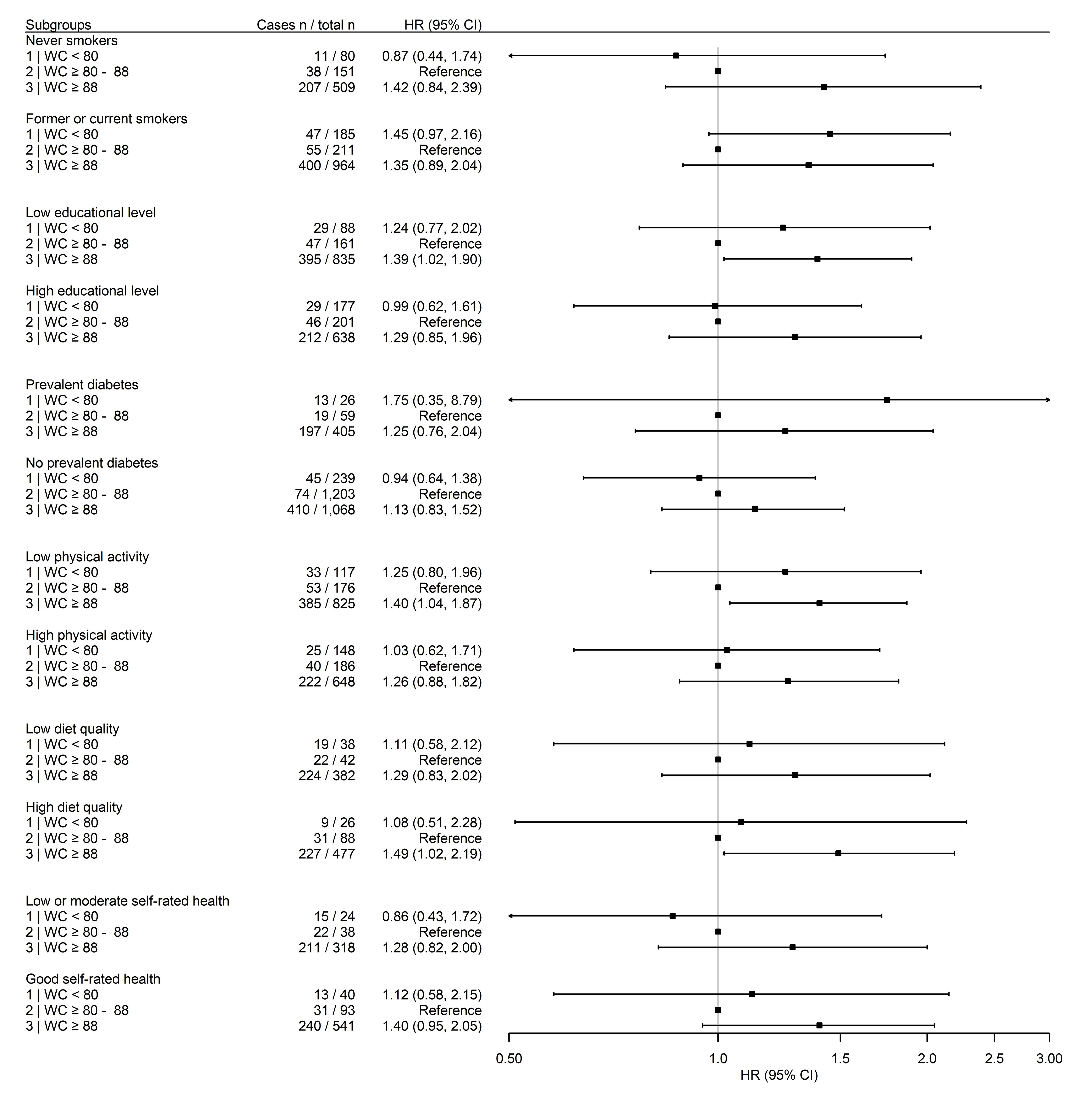

Supplement: S7 Fig — Associations were adjusted according to variables in model 2 unless for variable stratified for. Results for diet quality and self-rated health only available from AOC. (TIF) [file pone.0303329.s016.tif]
