# Supplementary material for: Association of body mass index and waist circumference with long-term mortality risk in 10,370 coronary patients and potential modification by lifestyle and health determinants
Source: PLoS One. 2024 May 31;19(5):e0303329. doi: 10.1371/journal.pone.0303329 (PMC11142547; doi:10.1371/journal.pone.0303329)
Supplement: Attachment — Submitted filename: Revision PLOS ONE draft0702_final.pdf [file pone.0303329.s017.pdf]

Comments from the editor

1. Please ensure that your manuscript meets PLOS ONE's style requirements, including those for file naming.

Author's reply: We carefully checked the style requirements and changed the manuscript accordingly.

2. Did you know that depositing data in a repository is associated with up to a 25% citation advantage. If you've not already done so, consider depositing your raw data in a repository to ensure your work is read, appreciated and cited by the largest possible audience.

Author's reply: Due to ethical reasons, the data cannot be made publicly available. However, we are open to collaboration and data sharing with other researchers. See the data availability statement for more information.

3. Thank you for stating the following financial disclosure:

"This work was supported by a grant from the Regio Deal Foodvalley (162135). The Alpha Omega Trial (2002–2009), from which this cohort study emerged, was supported by Netherlands Heart Foundation grant 200T401; NIH, National Heart, Lung, and Blood Institute, and Office of Dietary Supplements grant R01HL076200."

Please state what role the funders took in the study. If the funders had no role, please state: ""The funders had no role in study design, data collection and analysis, decision to publish, or preparation of the manuscript."" If this statement is not correct you must amend it as needed. Please include this amended Role of Funder statement in your cover letter; we will change the online submission form on your behalf.

Author's reply: We included the role of the funders in the financial disclosure: "The funders had no role in the study design, data collection and analysis, decision to publish or preparation of the manuscript."

4. Please include captions for your Supporting Information files at the end of your manuscript, and update any in-text citations to match accordingly.
Author's reply: Captions for the Supporting Information files were included at the end of the manuscript and citations are matched accordingly.

Reviewer 1

This manuscript of a prospective study is interesting because the authors proposed to evaluate the association of BMI and WC with mortality in CAD patients as well as their modifying effects. This well-written study also adds to the literature of mortality risk factors in CAD patients from two large cohorts. However, there are some important concerns to discuss, as follows.

Thank you for carefully reading our manuscript and for the valuable feedback.

Title:
1. The tittle should be replaced "body anthropometrics" with "body mass index and waist circumference" to be more specific and do not mislead readers.

Author's reply: We agree that the title could be more specific. We also incorporated the comments of the other reviewer and changed the title into the following:

> **Title page**
>
> Associations of body mass index and waist circumference with long-term mortality risk in 10,370 coronary patients and potential modification by lifestyle and health determinants

Methods:

2. Methods: How many patients with BMI <18.5 kg/m2 (underweight) were included? Patients with underweight and normal weight (BMI 18.5 – 25) could have a different association with mortality and could affect the results.

Author's reply: Of our total sample, <0.5% was underweight. Our continuous analysis indeed showed a higher risk of mortality in patients with a low BMI. In categorical analyses we combined underweight and normal weight patients in the same category. Leaving out the small number of underweight patients from our categorical analyses did not essentially change the results. We have added this information to the Results section.

> **Page 19, lines 293-294**
>
> Excluding patients with underweight (<0.5% of the study population) did not essentially change the results (data not shown).

3. To categorize levels of alcohol intake, why the amount of alcohol intake in each level differed between cohorts? Authors may covert amount of alcohol intake in drink to gram.

Author's reply: Alcohol intake was measured differently in the two studies. A food frequency questionnaire (FFQ) with detailed information on alcohol consumption (types, amounts) was used in the Alpha Omega Cohort. Based on that information, sex-specific categories were defined that showed strong associations with mortality (Cruijsen et al. Am J Clin Nutr 2022). In UCC-SMART, a general questionnaire was used that assessed alcohol consumption in standard drinks per weeks which was subsequently categorized. Alcohol was not the primary exposure of interest for the present study, but a confounder, and we decided to maintain the cohort-specific categorization that showed the best fit with the data, consistent with previous publications of both studies.

4. Please clarify why sex was used as covariate and stratification factor. Was there any interaction between BMI and sex, and WC and sex?

Author's reply: Body fat mass and fat distribution differ between men and women, as does mortality risk. In our combined analyses, we therefore adjusted for sex as a confounder. In addition, we performed stratification to obtain more insight in the role of sex, as a potential effect modifier. When designing the current study, we set an a priori goal to derive separate risk estimates for men and women, as well as other subgroups, independent of whether differences in risk estimates would be identified. There is lack of female-specific data in cardiovascular medicine. Our focus on group-specific risk assessment is crucial for the practical application of our findings, such as risk prediction models and communication to patients, which is why we do not formally test for (statistical) interaction. Results from subgroup

analyses should, however, be interpreted with caution. They form the basis for further study, rather than being statistical proof for group differences. This has now been emphasized in the Discussion section.

> **Page 23, lines 370 - 371**
>
> Subgroup results were not adjusted for multiple testing thus these results should be interpreted with caution. **They form the basis for further study rather than being statistical proof for group differences.**

Results and Discussion:

5. Page 11, Line 4: Unit (years) should be added after the numbers.

Author's reply: Done.

> **Page 11, line 240**
>
> Mean ages of patients were 69.0 ± 5.6 **years** in the AOC and 61.4 ± 9.5 **years** in the UCC-SMART.

6. For clarity, the percentage of CVD death and all-cause death should be presented. If the percentage is highly different, please discuss in this point.

Author's reply: The percentage of deaths in the Alpha Omega Cohort (47%) is higher than in the UCC-SMART cohort (23%) but this was mainly due to the age of participants at study enrolment. Patients in the Alpha Omega Cohort were already 60-80 years and patients in UCC-SMART were 18-79 years. We added the percentages of total deaths to our Results section.

> **Page 11, lines 216-217**
>
> During the follow-up period, a total of 2,287 deaths **(47% of total cohort)** occurred of which 1,010 were due to CVD in the AOC and a total of 1,266 deaths **(23% of total cohort)** occurred in UCC-SMART of which 610 were due to CVD.

7. Please carefully interpret the results from Figure 3. Some results cannot be addressed when the mortality risks were not statistically significant.

Author's reply: We agree that these results should be interpreted with caution. We are not primarily interested in statistical significance, but rather in the magnitude of risk estimates and risk patterns across subgroups. We went through the Results section belonging to Figure 3 and adjusted the phrasing in several places, showing more uncertainty.

Page 18, lines 279-285

For never smokers and patients with high physical activity, **a BMI <25 kg/m2 was not associated with a higher mortality risk.** ~~a J-shaped curve was observed where a BMI <25 kg/m2 was not associated with a higher mortality risk~~. A higher WC was consistently associated with a higher all-cause mortality risk compared to the middle category of WC (Supplementary Figure 3) especially in those with diabetes and high physical activity. A low WC was not significantly associated with mortality except for those with diabetes and low or moderate self-rated health showing **a trend towards** an increased mortality risk with low WC in these groups. Results for subgroup analyses further stratified for sex can be found in S4-7 Figs.

Table 1:

8. P-values among 3 BMI groups of each variable should be informed.

Author's reply: We adhered to STROBE principles (von Elm et al. PLOS Med 2007) and did not include p-values in the baseline table for the following reasons. 1) These p-values would only reflect univariate associations across one variable. However, most of the variables in the baseline table will be biased by other variables (e.g. sex and age) of the patients. 2) Statistically significant differences (two-sided P <0.05) in baseline characteristics are often used to select confounders. However, small differences in a confounder may still impact the associations and should also be adjusted for anyhow (Vandenbroucke et al. Ann Intern Med 2007).

9. Please correct Age (y) to Age, year and serum blood lipids to serum lipids

Author's reply: We agree, and this has been changed in Table 1.

Figure 1:

10. From RCS plots, female patients with BMI 25-30 kg/m2 seems to have lower mortality risk in UCC-SMART from plot whereas BMI >37 kg/m2 seems to have higher mortality risk. Please discuss in this point.

Author's reply: From the RCS plot, we can indeed observe that women from UCC-SMART with a BMI between 25-30 kg/m^2 have the lowest mortality risk. However, caution is warranted in interpreting results for BMI >30 kg/m^2 onwards, as the 95% confidence interval includes the value of 1.00. It appears that those with BMI >37 kg/m^2 may have a higher risk, but there is a large uncertainty around that estimate and we are hesitant to draw conclusions.

Figure 3:

11. P for interaction between subgroups of each lifestyle factor should be presented to confirm the difference between these subgroups.

Author's reply: As mentioned in our response to comment 4, our objective in this study was to obtain separate risk estimates within subgroups in order to focus on the practical application of our results in risk prediction models and communication to patients. Therefore we do not formally test for (statistical) interactions. Furthermore, more insight in potential effect measure modification can be obtained when the magnitude of the associations is considered, rather than evaluating a p-values.

12. Please be consistent with the word "participants" or "patients".
Author's reply: We consistently changed all wording of "participants" into "patients".

Reviewer 2

The study "Effect of lifestyle factors, education and indicators of health status on the association of body anthropometrics with long-term mortality in 10,370 patients with coronary artery disease" by E. Cruijsen et al. is theoretically and clinically important. However, several conceptual and methodological aspects of the paper should be clarified and the title and conclusions amended.

Thank you for carefully reading our manuscript and for the valuable feedback.

Introduction

1. The authors summarize: "The extent to which lifestyle behaviors or comorbidities such as diabetes might explain or modify the J- or U-shaped relationships between BMI and WC and mortality risk in CHD patients is not yet clear. The nature of the relationship might also differ between male and female patients (13) (Mohammadi et al. 2013 review: In the univariate analysis, the risk was increased in the fifth quintile (hazard ratio 1.22, 95% confidence interval 1.07-1.39) compared to the first. In the multivariable-adjusted analysis, the risk was increased in the fourth and fifth quintiles (hazard ratio 1.21, confidence interval 1.03-1.43 and hazard ratio 1.25, confidence interval 1.04-1.50, respectively). Sex-specific analyses showed similar associations in men, while U-shaped associations were observed in women and the body mass index analyses). The authors should clarify: What are the research hypotheses in this paper and control for multiple testing:

H1: There are J- or U-shaped associations between BMI and WC and the risk of death in CAD patients.
H2.there are gender-specific differences: linear correlations in men and U-shaped correlations in women
H3.lifestyle factors (smoking, physical activity, healthy diet) influence this association
H4.Socioeconomic status (SES)/education influences this association
H5.health status indicators affect this association

<u>Author's reply:</u> We agree with your line of reasoning and the hypotheses that were tested in this manuscript, in particular H1 and H2. However, our study of potential modifications by lifestyle and health determinants (H3, H4, H5) should be considered as exploratory. Insight in these modifications can be useful for risk stratification, risk prediction models and communication to patients. We did not define hypothesis for stratified analyses because we were only interested in the associations and risk patterns across subgroups. With respect to multiple testing, the results of our subgroup analyses form the basis for further study rather than being a statistical proof for group differences. We have rephrased several sentences in our Introduction and Discussion section.

Page 3, lines 58-68

To what extent **upstream (i.e. modifiable) lifestyle and health determinants** ~~behaviours or comorbidities, such as diabetes,~~ could explain or modify J- or U-shaped relationships of BMI and WC with mortality risk in CAD patients is not yet clear. The nature of the relationship may ~~also~~ differ between male and female patients. **Risk stratification for these determinants could provide** insight in the role of modifying factors ~~could~~ and guide health care professionals in identifying patients for weight loss therapy, and for personalised counselling **in weight management**~~. including optimal targets during weight loss interventions.~~ In the present pooling study of two prospective cohorts of CAD patients in the Netherlands, we performed sex-specific analyses of BMI and WC in relation to long-term risk of CVD and all-cause mortality. We additionally obtained insight in potential effect modification of these associations **by lifestyle and health determinants including** ~~lifestyle factors such as~~ smoking, physical activity, ~~and~~ diet quality, educational level, ~~and indicators of health status including~~ diabetes and self-rated health. **Insight in subgroup-specific associations can be useful for risk stratification and prediction, and risk communication to patients.**

Page 23, lines 370 - 371

Subgroup results were not adjusted for multiple testing thus these results should be interpreted with caution. **They form the basis for further study rather than being statistical proof for group differences.**

Methods

2. 4 837 CAD patients from the Alpha Omega Cohort (AOC) and 5 533 patients from the Utrecht Cardiovascular Cohort - Secondary Manifestations of ARTerial disease (UCC-SMART) were included in the study between 1996 and 2020. AOC was a randomized controlled trial of omega-3 fatty acids during the first three years of follow-up, while UCC-SMART focused on the treatment of cardiovascular disease. The patients were between 18 and 79 years old. Patients with a clinical diagnosis of CAD (defined as myocardial infarction, percutaneous coronary intervention, coronary bypass surgery and angina pectoris) were included. The study sample was therefore heterogeneous and was originally selected for other studies.

It is not clear whether a pre-planned secondary analysis was conducted.

Author's reply: All analyses were based on existing observational data. Regarding the Alpha Omega Cohort, patients had been randomized to low doses of omega-3 fatty acids during the first three years of follow-up. The intervention did not impact the risk of major cardiovascular events, and can be ignored as potential confounder (due to randomization). After the intervention, patients were followed for cause-specific mortality using national registers. We used a predefined analysis plan for this pooled cohort analysis, with the primary aim to study the association between BMI/WC and mortality and the potential modifying role of lifestyle and healthy determinants. Analyses like presented here are the main purpose of these observational cohort studies.

3. Dividing the sample according to clinical criteria into BMI <25; BMI 25-<30 and BMI ≥ 30 kg/m2 and comparing these three groups is, in my opinion, not statistically suitable to prove hypotheses 1-5 (see above). You need the entire sample for the calculation.

Author's reply: We agree that making use of the total study population for each analysis is preferred. For testing hypothesis 1 and 2 we used the entire sample in our restricted cubic splines analysis. Since this continuous analysis revealed non-linear associations, we cannot perform analysis in subgroups per increment in BMI or WC, assuming linearity of the association. To warrant comparability we decided to perform categorical analyses for the entire sample and across subgroups.

4. What are the lifestyle factor variables you want to examine in this study? Smoking, physical activity, alcohol consumption, diet quality?

Author's reply: Yes, indeed, these are the lifestyle variables that we included in our study. We focused on the upstream, modifiable determinants of BMI and waist circumference, which are relevant for prevention and personalized risk communication to patients. Other important lifestyle factors such as stress and sleep might also be relevant but both cohort studies lacked information on this.

5. Education as a proxy variable for SES should be explained.

Author's reply: We operationalized SES (also referred to SEP: socioeconomic position) as the highest level of education achieved. This was assessed through a questionnaire containing seven response categories, which were later aggregated into four categories for analysis (see Methods). We realize that the variable 'education' is only a proxy, since it does not cover all factors related to SEP, such as income and (former) occupation. Unfortunately, we did not have information on the latter in our cohort.

We have added the following information to our Methods and Discussion.

Page 6, line 97

Educational level **was used as a proxy for SEP and** was divided into four categories:

Page 22, line 348-349

Unfortunately, we did not have information on all factors related to SEP; therefore, we used educational level instead.

6. Measuring self-related health with a single item is very narrow and not suitable to prove H5. The DHD-CVD score consists of 16 items.

Author's reply: Even though self-rated health was assessed with one question, it showed significant predictive power for preterm mortality in our cohort of CAD patients. We agree that this variable does not capture the entirety of a patient's health. We have replaced "indicators of health status" with

“lifestyle and health determinants”. Alongside self-rated health, this denominator includes the presence of diabetes and lifestyle factors including smoking, physical activity and healthy diet.

We used the DHD-CVD index as measure of adherence to 16 dietary guidelines for cardiovascular patients, as formulated by the Health Council of the Netherlands (www.gr.nl/en). The index consists of 16 items which were summarized in a total score that was used for our analyses.

7. Further psychological measures of depression etc. are missing, as is the use of psychotropic drugs/antidepressants.
Author's reply: Including the use of psychological measures of depression is an interesting idea indeed. However, it was not part of our predefined analysis plan. It would also require specific knowledge on psychological and psychiatric disorder in CAD patients that we do not have in our team. We thank you for this interesting idea, and we may explore these and other interactions with medication in future studies.

8. Fig. 3-7 in the appendix provide no information for the hypotheses and can be omitted.
Author's reply: These figures in the supplementary material represent the results for waist circumference and mortality across subgroups (Figure 3) and sex-specific results (Figure 4-7). We deem these results clinically relevant for risk stratification and communication and they could be important for future meta-analyses. Particularly, data in women are relevant since they continue to be underrepresented in cardiovascular research. Especially data across subgroups in women is relevant since women remain to be underrepresented in cardiovascular research.

Discussion
9. The good performance of overweight men and women (BMI 25-30) is astonishing; what is the reason for this?
Author's reply: Better survival in CAD patients who are overweight or mildly obese is known as the ‘obesity paradox’, as observed also in other patient and elderly cohorts. This paradox may be attributed to physiological and methodological factors. Due to the observational nature of our study, we are unable to investigate specific underlying mechanisms, but we allude to them in the Discussion section.

Page 20, lines 307-312

Because of the observational nature of our study, we could not study underlying mechanisms to explain this paradox. However, physiologically, this discrepancy with **better survival in CAD patients** could be explained by better survival rates in overweight patients [30] and the protective effects of metabolic reserves **providing more resilience and protection** in times of illness or stress [31], especially during later stages of life as our population represents [32]~~., or the cardioprotective effect of certain adipokines produced by adipose tissue [2, 33]. Furthermore, general BMI guidelines are directed towards general morbidity and mortality prevention rather than solely premature mortality prevention.~~ Methodologically, this discrepancy could possibly be explained by collider stratification bias ~~that occurs when the qualifying event for inclusion in a study (CAD event) is a collider in the relationship between the exposure (obesity) and outcome (mortality)~~[34], reverse causation [35] or confounding by smoking [36], as discussed below.

10. And what are the reasons for the poor outcome of CAD patients with normal weight (BMI <25->18)? This is important for treatment guidelines for dietary habits in CAD patients, especially because a healthy diet had no effect on all-cause and CVD mortality.
Author's reply: Reasons for poor outcomes in normal weight CAD patients arise from the explanation raised in comment 9 on better outcomes in overweight patients.

Concerning the role of healthy diet, we concluded that this was not an effect modifier. We did not study healthy diet as an exposure. However, this was done previously in our cohort of CAD patients, showing a strong association between healthy eating and lower mortality risk (Sijtsma et al. Am J Clin Nutr 2015).

11. What is the reason for the U-shaped relationships between BMI and WC and mortality risk in CHD patients?
Author's reply: A U-shaped association between BMI/WC and mortality is consistently observed across different populations, including generally healthy people (Visaria et al. PLOS ONE, 2023). Due to the abovementioned reasons (comment 9), this U-shape is shifted to the right. Higher mortality risk for low BMI may be explained by smoking and its related diseases, frailty, (pre)clinical cancers and acute disease (Jorgensen et al. Obesity, 2016). Higher mortality risk at the higher range (obesity) is likely due to cardiometabolic and other non-communicable disease.

Page 20, line 305-306

Current guidelines for the general population recommend a BMI between 18 and 25 kg/m2 for optimal health, but the optimal BMI appears to be shifted to the right in CAD patients. ~~These findings contrast with current recommendations for the general population, for which a BMI between 18.5 and 25 kg/m2 is considered most optimal for health.~~

12. What are the reasons for the sex difference?
Author's reply: Possible reasons for the observed sex differences could be the distribution of adipose and muscle tissue and its impact on cardiovascular health for both men and women. However, our observational data do not provide causal relationships, but form the basis for group-specific risk assessment. Sex-specific differences should be interpreted with caution due to relatively small sample of women.

13. Why does all-cause mortality show this U-shaped relationship, but CVD mortality does not? One might expect the opposite.
Author's reply: In our view, U-shaped relationships are seen both for all-cause and CVD mortality, especially in men. In women from the Alpha Omega Cohort, a linear association was found but these results should be interpreted with caution because of the small sample size.

14. Diabetes, hypertension, LDL cholesterol, CRP, history of MI or CABG, and heart failure (NYHA, EF) are important physical modifiers of CVD mortality and all-cause mortality and should be discussed in more detail for the association between obesity/overweight/not overweight/not obese and mortality.
Author's reply: As mentioned in comment 4, this research focuses on upstream, modifiable

determinants of BMI and waist circumference including lifestyle and other health determinants. By focusing on upstream determinants like smoking, physical activity and healthy eating, personalized practical advice and counselling could be given to patients by health care providers based on these determinants. We changed the Introduction for clarification.

Page 3, line 58

To what extent **upstream (i.e. modifiable)** lifestyle and health determinants could explain or modify J- or U-shaped relationships of BMI and WC with mortality risk in CAD patients is not yet clear.

15. The limitations of the study results (see above) should be discussed in more detail.
Author's reply: The abovementioned comments were considered for discussion in the limitations. However, we believe that our limitations section in the Discussion sufficiently covers the limitations that accompany our study. As mentioned in comment 1 we added the following to the limitations in the Discussion.

Page 23, lines 370 - 371

Subgroup results were not adjusted for multiple testing thus these results should be interpreted with caution **as they form the basis for further study rather than being statistical proof for group differences.**

Page 23, lines 376-377

Further, certain variables, such as self-rated health and SEP were not fully captured due to limitations of the collected data.

Conclusion

16. The authors' statement: "Whether overweight is causally associated with a lower risk of premature mortality in CAD patients remains uncertain due to the limitations of this observational study. Obesity prevention ..... should be promoted in all CAD patients" cannot be accepted as overweight has the lowest mortality risk and should not be treated. Grade II and III obesity in CAD patients can be treated.
Author's reply: We adapted and shortened the conclusion to better fit our findings, we additionally adapted corresponding sentences in our manuscript like the first paragraph of the Discussion and Abstract.

Discussion

In summary, we found U-shaped associations for BMI and WC with long-term CVD and all-cause mortality risk in CAD patients. Patients with obesity and a large WC were at increased mortality risk, as were underweight patients, while mildly overweight patients (BMI ~ 27 kg/m2) had the lowest mortality risk. These U-shaped associations persisted across diverse subgroups of CAD patients characterized by varying lifestyle factors and health status. Our findings may have implications for risk stratification and effective risk communication to CAD patients. ~~To summarize, we observe the largest risk reduction of all-cause mortality and CVD mortality for a BMI of 25-30 kg/m2 in CAD patients. For WC a similar pattern was observed, with the lowest mortality risk in those with an intermediate WC. Excess body fat, indicated by a high BMI and WC was associated with higher risks of premature mortality independent of diet quality, self-rated health and diabetes, while physical activity, educational level and smoking seem to modify this association to some extent. Whether being overweight is causally related to a lower risk of premature mortality in CAD patients remains uncertain due to limitations related to this observational study. Obesity prevention, smoking cessation and engaging in sufficient physical activity should be encouraged in all CAD patients.~~

17. The title of this paper: "Influence of lifestyle factors, education and indicators of health status on the association of anthropometric body measures with long-term mortality in ...patients with coronary heart disease" is misleading because the calculation of the data did not produce these results.

Author's reply:

Based on the comments of the reviewers we adapted the title of our manuscript into the following. We believe that this title better reflects the contents of our manuscript.

Title page

Association between body mass index, waist circumference, and long-term mortality in 10,370 coronary artery patients and potential modification by lifestyle and health determinants.
