# Supplementary material for: Association of body mass index and waist circumference with long-term mortality risk in 10,370 coronary patients and potential modification by lifestyle and health determinants
Source: PLoS One. 2024 May 31;19(5):e0303329. doi: 10.1371/journal.pone.0303329 (PMC11142547; doi:10.1371/journal.pone.0303329)
Supplement: Attachment — Submitted filename: Response to reviewers R2.pdf [file pone.0303329.s018.pdf]

Response to the reviewers

Journal requirements:

Please review your reference list to ensure that it is complete and correct. If you have cited papers that have been retracted, please include the rationale for doing so in the manuscript text, or remove these references and replace them with relevant current references. Any changes to the reference list should be mentioned in the rebuttal letter that accompanies your revised manuscript. If you need to cite a retracted article, indicate the article's retracted status in the References list and also include a citation and full reference for the retraction notice.

Author's reply: We carefully reviewed our reference list and it is complete and correct. We checked for any possible retracted papers using Retraction Watch, confirming that none of our included references has been retracted.

Reviewer 2

The manuscript is much better now. I have only two points where I am not satisfied with the authors' response.

Author's reply: Thank you for reviewing our revised manuscript. We hope to have adequately addressed your raised comments now.

1. The authors have cited the work of Mohammadi et al (2013) who performed a univariate analysis and found that the all-cause mortality risk was increased in the fifth quintile compared to the first quintile. In the multivariable-adjusted analysis, the risk was increased in the fourth and fifth quintiles. Why did the authors not use this type of calculation in each substudy separately and in the combined study to avoid the somewhat artificial clinical BMI classification?

Author's reply: The article of Mohammadi et al (2020) used quintiles to categorise waist circumference and they used three groups (normal weight, overweight, obesity) to categorise BMI. Besides our clinical BMI and WC categorisation in three groups, we used restricted cubic splines to model continuous associations between BMI and WC with mortality risk. This method uses all available datapoints for BMI or WC rather than a categorisation of the data. By using these two methods, we believe we have adequately captured the (non-linear) associations between BMI and WC with mortality .

2 The authors stated: "We did not define hypotheses ... because we were only interested in the associations and risk patterns between the subgroups" and "our study on possible changes by lifestyle and health factors (H3, H4, H5) should be considered exploratory."
My response: if they don't have research hypotheses, they shouldn't publish their data, but they agreed to my suggestion related to two hypotheses that could be answered with their data:

"H1: There are J- or U-shaped associations between BMI and WC and risk of death in CAD patients.
H2: There are gender differences: linear correlations in men and U-shaped correlations in women"
If the authors agree, exploratory research with less statistical power could describe these associations:

1.Lifestyle factors (smoking, physical activity, healthy diet) influence this association
2.socioeconomic status (SES)/education influences this association
3.health status indicators influence this relationship

This structure of the research paper should be given at the end of the introduction.

Author's reply: We agree that our analyses on lifestyle factors, educational level and health status indicators should be regarded as exploratory. We revised the sentence outlining the structure of the manuscript to emphasize that these analyses are indeed exploratory.

Based on comments from other reviewers, we redefined our exploratory factors as lifestyle and health determinants, and we delineate all determinants in our introduction.

Introduction, line 65

~~We additionally obtained~~ **Exploratory analyses were performed to obtain** insight in potential effect modification of these associations by lifestyle and health determinants including smoking, physical activity, diet quality, educational level, diabetes and self-rated health.

3. In my opinion, omitting several tables in the appendix would have made the structure of the paper clearer, but if the authors want to keep them in the paper, they should do so.

Author's reply: As mentioned in our previous response, we are indeed in favour of including the supplementary tables for risk stratification, communication and future meta-analyses.
